# Factor structure and psychometric properties of a Polish adaptation of the Warwick–Edinburgh Mental Wellbeing Scale

**DOI:** 10.1186/s12955-021-01716-w

**Published:** 2021-03-02

**Authors:** Karol Konaszewski, Małgorzata Niesiobędzka, Janusz Surzykiewicz

**Affiliations:** 1 Faculty of Education, University of Bialystok, Bialystok, Poland; 2 Faculty of Philosophy and Education, Katholische Universität Eichstätt-Ingolstadt, Eichstätt, Germany; 3 Faculty of Education, Cardinal Stefan Wyszynski University in Warsaw, Warsaw, Poland

## Abstract

**Background:**

The study of mental wellbeing requires reliable, valid, and practical measurement tools. One of the most widely used measures of mental wellbeing is the Warwick–Edinburgh Mental Wellbeing Scale (WEMWBS). We conducted four studies to validate the Polish version of the WEMWBS. Their objectives are the following: (1) to present the psychometric properties of the Polish version of the WEMWBS (study 1: n = 1197); (2) to evaluate the test–retest reliability of the Polish version of the WEMWBS (study 2: n = 24); (3) to determine the validity of the WEMWBS (study 3: n = 610); (4) to examine sensitivity of the WEMWBS scale to detect population with different levels of pro-health behaviours (study 4: n = 430).

**Methods:**

To explore the dimensional structure of the scale we tested a one-factor model. The evaluation employed explanatory and confirmatory factor analyses and tested reliability and stability. To determine the convergent validity of the WEMWBS we analysed correlations among wellbeing and life satisfaction and risk depression. To examine sensitivity of the WEMWBS scale to detect a population with different levels of health-related behaviours we used Student’s t test.

**Results:**

The results presented confirm that the psychometric properties of the Polish adaptation of WEMWBS are very good. Using EFA and CFA it was shown that a one-factor solution is optimal. Reliability, measured using the Cronbach’s alpha coefficient and McDonald's omega proved to be very high. The estimation of the stability of the Polish version of the WEMWBS proved to be high. Our validation studies also provided data demonstrating sensitivity of the WEMWBS to detect a population with different levels of health-related behaviours, indicating that group with high level of pro-health behaviours achieved higher WEMWBS wellbeing results than group with low level of pro-health behaviours.

**Conclusions:**

WEMWBS was confirmed as a short, reliable and valid measure with good psychometric properties. Due to the high indicators for its psychometric properties, the scale may therefore prove to be a particularly useful tool not only in empirical research, but also in mental wellbeing monitoring, and could serve as support in educational and preventive.

## Introduction

Theoretical solutions and research indicate that mental wellbeing is probably best seen as a multidimensional phenomenon involving aspects of both the hedonic and the eudaimonic concepts of wellbeing [1–3]. Stewart-Brown has combined two aspects of wellbeing. In this approach mental wellbeing is a concept regarded as encompassing dimensions of hedonic (positive feelings, affect, emotions) and eudaimonic (positive functioning, mindset and relationships) wellbeing [4–9]. This form of wellbeing is achieved through the self-development of character traits and behaviour [9–11].

Most tools for measuring aspects of mental health are focused on negative areas, illness and suffering [1, 12, 13]. In contrast, respondents and patients prefer ‘positive’ tools [14]. As a result, ‘positive’ tools provide better support for building positive psychological interventions. Furthermore, the aim of these tools is to measure a specific aspect of mental health, such as emotions (Positive and Negative Affect Schedule; PANAS) [15], cognitive evaluation (Satisfaction with Life Scale; SWLS) [16], or eudaimonic well-being (Psychological Well-being Scales; PWBS) [17]. Stewart-Brown and colleagues designed a research tool, namely the Warwick–Edinburgh Mental Well-being Scale (WEMWBS), to capture the wide concept of well-being [5, 7]. The scale includes affective-emotional aspects, cognitive-evaluative dimensions and psychological functioning as well. The WEMWBS scale is a self-describing measure of mental wellbeing that includes both eudaimonic and hedonic dimensions, and is focused exclusively on positive aspects of mental health. WEMWBS assesses both hedonic (positive emotions, happiness, joy, interest and contentment) and eudaimonic (psychological functioning associated with personal growth, autonomy, self-acceptance, mastery, positive relationships with others, and a sense of purpose in life) mental wellbeing. It was developed to enable the measurement of mental wellbeing in the general population and the evaluation of projects, programmes and policies that aim to improve mental wellbeing. The items are all worded positively and cover both the feeling and the functioning aspects of mental wellbeing, thereby making the concept more accessible. The scale has been widely used nationally and internationally for monitoring and evaluating projects and programmes, and investigating the determinants of mental wellbeing [5, 7, 18].

In addition, the authors of the tool pointed to the need to develop a research tool that could be used in psychological and medical areas, as well as in social policy. There was a demand from those interested in public mental health for a measure suitable for monitoring mental wellbeing that did not show ceiling effects in population samples. There was also a demand from specialists in the promotion of mental health for a tool with which they could evaluate their programmes. Activities with a negative attitude may suggest to participants that such programmes are intended for people with mental health problems, and thus diminish and fail to support these initiatives. Based on the Affectometer 2, the academic literature and nine focus group interviews, an expert panel developed WEMWBS in 2006. This scale aims to build on previous scales and to capture a wide conception of wellbeing in a form that is short enough to be used in population-level surveys. By focusing wholly on the positive, the scale is intended to support mental health promotion initiatives and be free of ceiling effects [5, 7, 10, 18].

WEMWBS shows good content validity. Confirmatory factor analysis (CFA) supports the single factor hypothesis. A Cronbach’s alpha score of 0.89 (student sample) and 0.91 (population sample) suggests some item redundancy in the scale. Its distribution is near normal and the scale did not show ceiling effects in a population sample. Test–retest reliability at 1 week was high (0.83) [5, 7, 18]. WEMWBS covers the main concepts associated with positive mental health, for example: positive affect, satisfying interpersonal relationships and positive functioning [5, 6, 10, 19].

WEMWBS has been translated into a number of languages, and some of these translations have been validated both psychometrically and qualitatively. A one-factor solution has been confirmed in many research [20–25]. The validity of WEMWBS has most often been determined based on a criterion for constructs measuring personality traits such as self-esteem or mental and physical health results. Positive correlations have been noted between mental wellbeing and self-esteem, life satisfaction, positive affect, and wellbeing scales such as WHO-5, and negative correlations between mental wellbeing and mental health problems (such as depression and anxiety), mental disorders, school pressure and bullying victimisation [5, 20, 22, 23, 26]. WEMWBS is characterized by good psychometric properties, and it is economical in relation to the number of items, which are positively formulated. The scale has been used in different populations and has been translated into many languages. We therefore chose to adapt WEMWBS. Therefore, the validation of a proven and valuable tool in this field—that is, WEMWBS—under Polish conditions will help to develop an important conceptual and diagnostic research area. Despite the good psychometric properties of the WEMWBS, recent literature has indicated some problems related to the items’ hierarchy and the existence of gender-biased questions [27]. In response, a shortened version of the WEMWBS (called the SWEMWBS) has been proposed and validated [20, 26, 28–31]. Fung [32] has demonstrated that the SWEMWBS possesses better construct validity than the full version of the WEMWBS and thus the purpose of our next study will be to create a Polish version of the SWEWBS.

### Study objective: Polish validation of the Warwick–Edinburgh Mental Wellbeing Scale

**Fig. 1:**
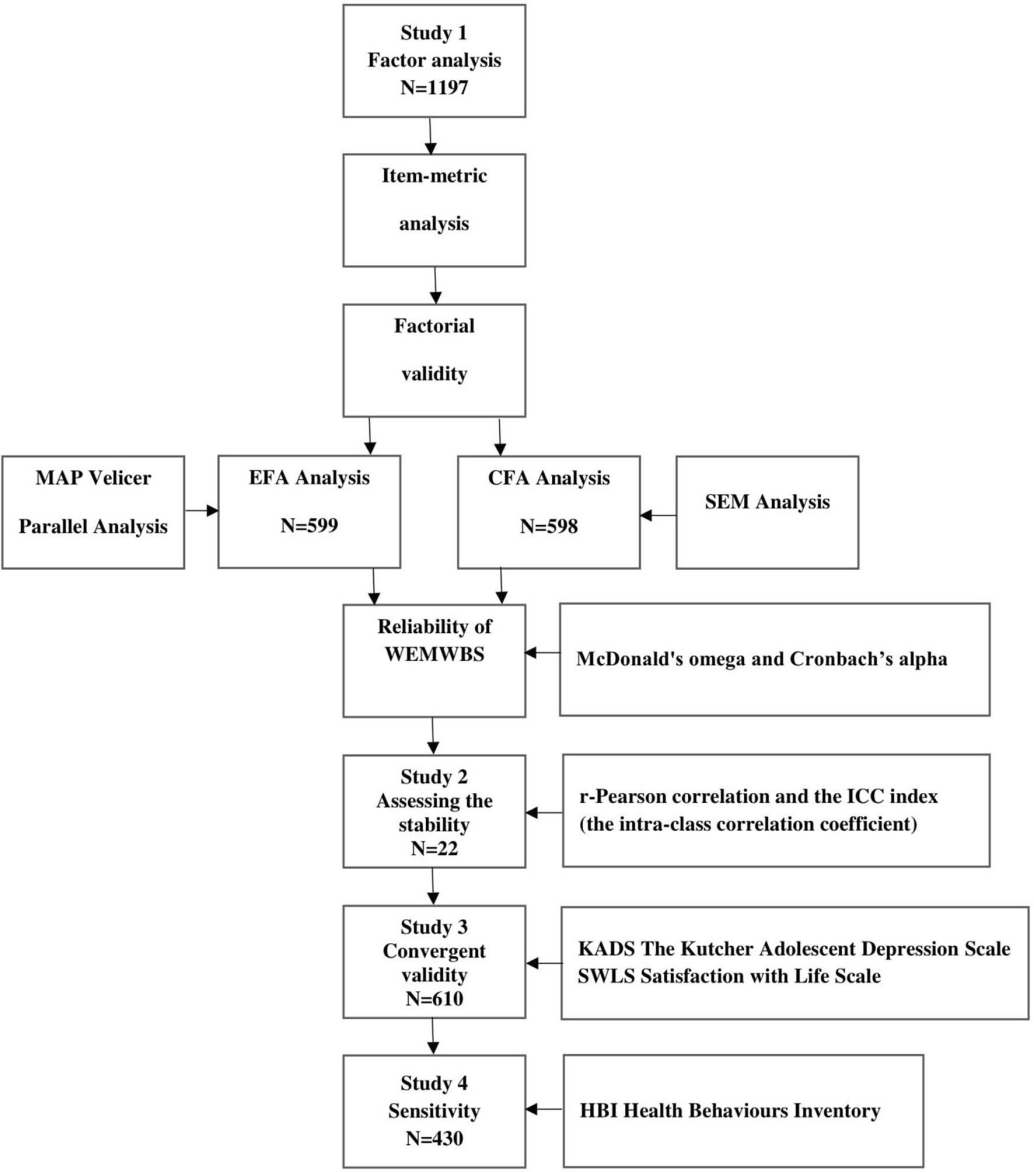
Development and validation process of the Polish version of the WEMWBS

The purposes of the research presented here were to adapt the WEMWBS into Polish and to estimate its psychometric properties. We conducted four studies to validate the Polish version of the WEMWBS. The first study presents the factorial validity and reliability of the Polish version of the WEMWBS (study 1: n = 1197). The second study demonstrates the stability of the Polish version of the WEMWBS (study 2: n = 24). The third study presents the convergent validity of the WEMWBS (study 3: n = 610). In line with the previous results, we expected the WEMWBS to have a positive relationship with life satisfaction [7] and a negative relationship with risk of depression [33, 34]. The fourth study demonstrates the sensitivity of the WEMWBS to detecting populations with different levels of pro-health behaviours (study 4: n = 430). In line with Stewart-Brown and colleagues’ results [10, 26, 35], we expected that the group with a high level of health-related behaviours would score higher on well-being than the group with a low level of health-related behaviours. The entire process of developing and validating the WEMWBS is presented in Fig. 1.

## Study 1: Exploratory factor analysis (EFA) and confirmatory factor analysis (CFA)

### Materials and methods

#### Participants

1197 participants aged between 20 and 63 took part in the validation study (*M* = 25.69; *SD* = 6.23). The group were predominantly women (69%). There were similar percentages of single people (46.7%) and people in a relationship (44.6%); only 8.4% of the sample were married, while the percentage of people who were divorced was 0.2%. No clear differences were determined in terms of educational and professional situation – people working and studying at the same time constituted 39.5% of the respondents, and people who were just studying constituted a similar percentage (34.1%). Respondents who worked made up 22.6% of the sample, while 3.9% of the sample were neither studying nor working at the time of the study.

#### Procedure and ethics statement

The research was carried out through direct contact with the respondents (n = 211) and via the Internet (n = 986). We recruited the sample among full-time and part-time students and alumni of the University of Bialystok. Full-time students in the Faculty of Education filled out the Polish version of the WEMWBS during a lecture. Having received information about the research, data confidentiality, the voluntary nature of participation and the possibility of withdrawing from the research at any moment, the respondents provided their informed consent. The online procedure was very similar: the full-time (except for the students in the Faculty of Education) and part-time students and alumni of the University at Bialystok were sent an email explaining the purpose of the research and data confidentiality and informing them that participation was voluntary and that they could withdraw at any time. The message also contained a link to the online questionnaire and access passwords to complete it, once participants had provided their informed consent. Ethical approval for the research project involving four studies was granted by the Ethics Committee of the Education Faculty at the University of Bialystok (ethical approval number: 03/2020). The data was collected over a 2 months period, from January to February 2020. All the participants were adults, and gave their written consent to participate in the research in accordance with the Helsinki Declaration.

#### Measure

##### The Warwick–Edinburgh Mental Wellbeing Scale (WEMWBS)

WEMWBS is formed by 14 items with a 5-point Likert response scale ranging from 1 = none of the time to 5 = all of the time. The global score ranges from 14 to 70. The higher the global score, the higher the level of mental wellbeing. The original version of the WEMWBS demonstrated good internal consistency with a Cronbach’s alpha value ranging from 0.89 to 0.91. Test–retest reliability at 1 week was high (0.83) [7].

##### Polish version of WEMWBS

Development of the Polish language version of the WEMWBS was prepared with support in several stages from author of the tool, Stewart-Brown. The scale was translated into Polish in line with WHO recommendations, which includes (1) forward-translation, (2) expert panel back-translation, (3) pre-testing and cognitive interviewing, and (4) final version. After the author’s consent to prepare a Polish version of the WEMWBS, the tool was translated into Polish by two independent translators under the supervision of a person with knowledge of psychological research methodologies. Then, in order to identify inappropriate translation statements as well as possible discrepancies, a panel of experts was put together, composed of principal investigators, the original translator and two experts in health psychology. Then, following the same approach as in the first stage, the tool was translated back into English by an independent translator whose mother tongue was English and who did not know the questionnaire. Efforts were also made to ensure that the Polish-language version would be well suited to the ages of the people representing the population for whom the tool was being translated. For this reason, the Polish-language version of the scale was evaluated in a group of 10 people aged 18 to 60. The translated version of the tool was provided during a group interview. The respondents were asked to mark “yes” if a question was completely understandable and “no” if it was incomprehensible or if it raised any doubts. Ultimately, every question was marked as understandable to the respondents. The calculated content validity ratio was 0.91, as also estimated by Kendall’s W test, indicating the considerable clarity and comprehensibility of the analyzed construct. The final Polish version of the WEMWBS was approved by a panel of experts.

#### Statistical analysis

All statistical calculations were performed using IBM SPSS Statistics 25 and JASP. The validation analyses were initiated with item-metric analyses, in which the questionnaire’s statements were analysed. Factor accuracy was checked by and exploratory factor analysis (EFA) using the principal component method with oblimin rotation. Before performing the analysis, the number of optimal components to extract was checked using the Velicer’s minimum average partial (MAP) method (based on original 1976 and revised 2000 version [36, 37]). A factor structure was also confirmed with Parallel Analysis (PA). Confirmatory factor analysis (CFA) with diagonally weighted least squares (DWLS) estimation in JASP was applied to verify the factor structure of the measures. A range of goodness-of-fit statistics and the appropriateness of the model parameters evaluated the overall model fit: the chi-square statistic, comparative fit index (CFI) and the goodness-of-fit index (GFI) and the root-mean-square error of approximation (RMSEA) [38–40]. EFA and CFA carried out with randomly split samples in half. The reliability of the scale was tested using Cronbach’s alpha coefficient; in addition, the values of the discriminatory power for individual items were obtained. Furthermore, for the reliability, we calculated McDonald's omega ω as an indicator less burdened with assumptions than Cronbach’s alpha coefficient [41].

### Results

#### Item-metric analysis

Table 1 show the basic descriptive statistics for the analysed items and the IDI (Item Difficulty Index) value of WEMWBS. The distribution of the analysed items did not differ significantly from the normal distribution. In addition, the IDI did not show the existence of floor or ceiling effects in the data. A frequency analysis for individual test items showed no problems with data granulation. For that reason, all items were included in the further analyses.

**Table 1:** Descriptive statistics and IDI value for individual items of WEMWBS

	M	SD	Skewness	Kurtosis	IDI
item1	3.52	0.97	− 0.36	− 0.40	0.70
item2	3.57	1.01	− 0.45	− 0.26	0.71
item3	3.12	0.93	− 0.20	− 0.40	0.62
item4	3.58	1.02	− 0.40	− 0.39	0.72
item5	3.31	0.99	− 0.30	− 0.36	0.66
item6	3.46	1.02	− 0.49	− 0.21	0.69
item7	3.71	0.92	− 0.52	− 0.05	0.74
item8	3.50	1.08	− 0.47	− 0.46	0.70
item9	3.49	1.04	− 0.42	− 0.42	0.70
item10	3.37	1.09	− 0.36	− 0.58	0.67
item11	4.00	0.92	− 0.80	0.29	0.80
item12	3.75	1.12	− 0.63	− 0.40	0.75
item13	4.02	0.98	− 0.96	0.56	0.80
item14	3.47	0.97	− 0.41	− 0.20	0.69

#### Factorial validity

A Velicer’s minimum average partial (MAP) analysis determined a one-factor structure for the WEMWBS. The smallest average squared partial correlation was 0.0211 (Table 2).

**Table 2:** Average squared partial correlation for individual items of WEMWBS

Root	Average squared partial correlation
0	.2155
1	.0211
2	.0222
3	.0273
4	.0390
5	.0572
6	.0781
7	.0969
8	.1263
9	.1606
10	.2193
11	.3039
12	.4858
13	1.000

**Fig. 2:**
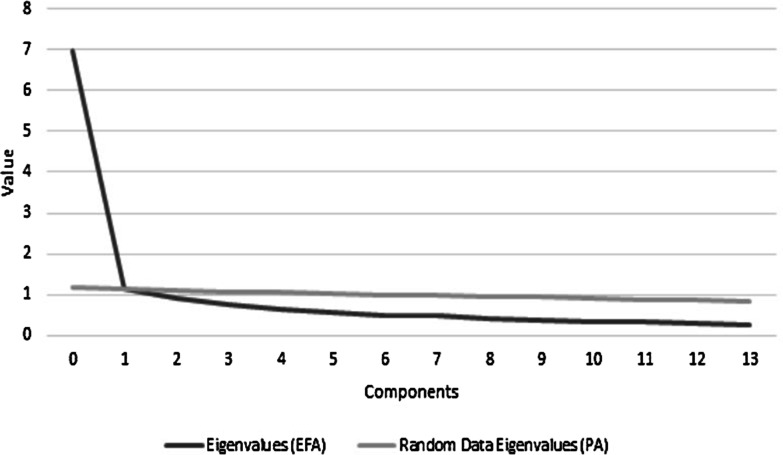
Comparison of EFA and PA results

A factor analysis using the principal components method with oblimin rotation also showed that the created component explains 48.98% of the analysed construct (KMO = 0.93; *chi*^2^(91) = 4237.15; p < 0.001). The factor loadings of EFA were high, from 0.56 to 0.82. A single-factor solution was also confirmed using parallel analysis (PA, principal components). The principal components and eigenvalues were based on all of the variance in the correlation matrices, including both the variance shared among the variables and the variances unique to them. A comparison of the eigenvalues from both analyses (EFA and PA) is presented below (Fig. 2).

**Fig. 3:**
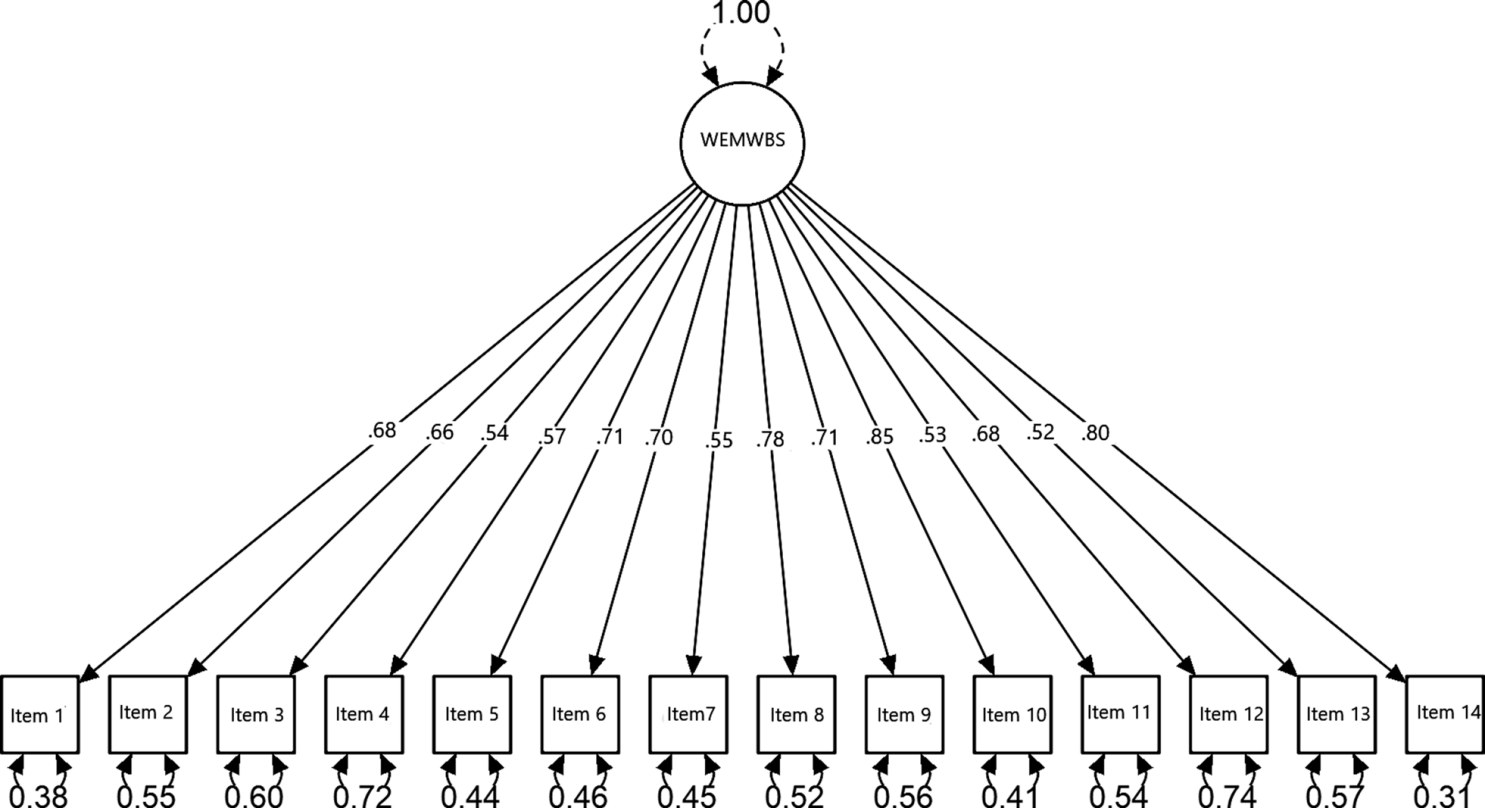
Single-factor structure of the Polish WEMWBS version

The model was also verified by confirmatory factor analysis with diagonally weighted least squares (DWLS) estimation. The created model had very good fit properties (*χ*2(77) = 167.97; *p* < 0.001*; χ*2*/df* = 2.18.; GFI = 0.98, CFI = 0.98, RMSEA = 0.044 [0.035–0.054]). The factor loadings were high and exceeded a magnitude of 0.52. Figure 3 demonstrates the standardized estimates of the confirmatory model.

#### Reliability of the WEMWBS

Cronbach's alpha coefficient demonstrated very good reliability of the WEMWBS, with α = 0.92. The composite reliability was also very good, with McDonald's omega ω = 0.91, which indicates the proportion of a scale’s variance due to a unidimensional factor.

#### Descriptive statistics of the indicators

The index obtained had values ranging from 14 to 70 points. The average for the overall result was *M* = 49.87 (*SD* = 9.88). The distribution of the index does not differ significantly from the normal distribution (skewness = − 0.45; kurtosis = 0.09). Analysis by t test for independent samples did not confirm that there was any relationship between the results obtained on the mental wellbeing scale and sex (*t(582*) = 0.08; *p* = 0.938). The *r*-Pearson correlation showed a low, positive correlation between the results on the wellbeing scale and age (*r* = 0.12; *p* < 0.001). Because of the low value of the correlation effect (0.016) and the large sample (N = 1197), age was not included in the further validation analyses.

## Study 2: Assessing the stability of the WEMWBS

### Materials and methods

#### Participants

The stability assessment was carried out using a group of 24 (Age: *M* = 21.67; *SD* = 0.89) social worker students of the Faculty of Education at the University of Bialystok. Most of the participants were women (54.2%). Students filled out the WEMWBS during the lecture in March 2020.

#### Materials

##### The Warwick–Edinburgh Mental Wellbeing Scale

The Polish version of WEMWBS tested in the study 1 was used to assess mental wellbeing.

#### Statistical analysis

The aim of the second study was to verify the stability of the WEMWBS. Stability was confirmed with the *r*-Pearson correlation and the ICC index (the intra-class correlation coefficient). A second test was performed with the same tool (the WEMWBS) 2 weeks after the first test. In order to verify the stability of the adapted tool, a Student’s *t* test was carried out for the dependent tests, and the *r*-Pearson correlation and the ICC index were used.

### Results

The Student’s *t* test analysis for dependent samples showed that the results of the respondents did not differ significantly in the second measurement, which shows that the scale is stable; *t*(23) = − 0.25; *p* > 0.05. The coefficient obtained for absolute stability (correlation between the test and the retest) proved that there was a high level of time stability for the WEMWBS results; *r*(24) = 0.87; *p* < 0.001. High stability was also confirmed by the ICC index; using the absolute compliance method, a high ICC was observed, ICC = 0.930; *F*(23, 23) = 14.27; *p* < 0.001. Over a short period of time, the measurement of mental wellbeing with the WEMWBS is characterized by a relatively high repeatability.

## Study 3: Test convergent validity of WEMWBS

### Materials and methods

#### Participants and procedure

Study 3: The sample participants were 610 students of the Education Faculty at University of Bialystok, aged from 20 to 24 years (age *M* = 22.52, *SD* = 1.10), including 491 women (80%) and 119 men (20%). Students received a link to the online questionnaire and access passwords to complete the questionnaire once they had provided informed consent. The individual questionnaires in the questionnaire package were arranged in the following order: WEMWBS first, Kutcher Adolescent Depression Scale (KADS) second and Satisfaction with Life Scale (SWLS) third. The study was conducted in March 2020.

#### Measures

##### The Warwick–Edinburgh Mental Wellbeing Scale

The Polish version of WEMWBS tested in the study 1 was used to assess mental wellbeing. Confirmatory factor analysis showed the good fit of the one-factor model to empirical data: (*χ2* (*77*) = 177.24; *p* < 0.001*; χ2/df* = 2.30; GFI = 0.98, CFI = 0.98, RMSEA = 0.046 [0.037 – 0.055]).

##### Satisfaction with life

Life satisfaction [16] “was measured with The Satisfaction with Life Scale (SWLS), developed by Diener and colleagues and adapted by Juczynski (1999), which assesses the cognitive aspect of SWB. The SWLS consists of five items rated by a respondent using a seven-point scale, ranging from ‘strongly disagree’ (1) to ‘strongly agree’ (7). Items are summed to give a total score ranging from 5 (low satisfaction) to 35 (high satisfaction). Sample items include “I am satisfied with the conditions of my life” and “So far, I have gotten the important things I want in life.” The Polish version of the SWLS had shown test–retest reliability (0.86), internal consistency—Cronbach's alpha (0.81), and discriminant validity (up 0.50)” [42].

##### Risk of depression

The Kutcher Adolescent Depression Scale (KADS) [43] “is a commonly used screening test used to identify young people at risk for depression. It is a self-report scale specifically designed to diagnosis and assess the severity of adolescent depression. The KADS consists of six items (sadness, lack of faith, physical exhaustion, sense of hardness of life, worries, and suicide of thoughts) rated by a respondent using a four-point scale, ranging from ‘hardly ever’ (0) to ‘all of the time’ (3). Total score ranged from 0 to 18. Validation of the Polish version of KADS in a group of students aged 18–24 years has shown its good reliability (0.82) and content validity [12].

#### Statistical analysis

The aim of the third study was to evaluate the validity of the Polish version of the WEMWBS. The validity was determined using r-Pearson correlation analysis to establish the relationships between mental wellbeing and life satisfaction and risk of depression.

### Results

To assess for validity support in the sample, correlations were calculated between the mental wellbeing scale (WEMWBS) and the satisfaction-with-life scale (SWLS), as well as with the risk of depression scale (KADS). As expected, mental wellbeing strongly and positively correlated with life satisfaction (*r* = 0.66, *p* < 0.001) and strongly and negatively with risk of depression (*r* = − 0.65, *p* < 0.001). Strong negative correlations were observed especially between mental wellbeing and lack of faith (r = − 0.61, p < 0.001) and sense of hardness of life (r = − 0.58, p < 0.001). Table 3 demonstrates the correlations between WEMWBS and SWLS and KADS including their dimensions.

**Table 3:** Pearson’s Correlations between the Variables (*N* = 610)

	1	2	3	4	5	6	7	8	9
1. Mental-Wellbeing	–								
2. SWLS	.668^**^	–							
3. KADS	− .659^**^	− .522^**^	–						
4. Sadness	− .555^**^	− .434^**^	.855^**^	–					
5. Lack of faith	− .610^**^	− .487^**^	.865^**^	.731^**^	–				
6. Physical exhaustion	− .492^**^	− .402^**^	.796^**^	.620^**^	.586^**^	–			
7. Sense of hardness of life	− .584^**^	− .476^**^	.856^**^	.660^**^	.690^**^	.670^**^	–		
8. Worries	− .529^**^	− .394^**^	.801^**^	.620^**^	.615^**^	.550^**^	.617^**^	–	
9. Suicide of thoughts	− .316^**^	− .253^**^	.532^**^	.378^**^	.450^**^	.261^**^	.348^**^	.317^**^	–

^**^p < .001

## Study 4: Sensitivity of the WEMWBS

### Materials and methods

#### Participants and procedure

The sample consisted of 430 students of the Faculty of Education at the University of Bialystok, aged 20–29 years (*M* = 22.35, *SD* = 2.21). Most of the participants were women (78%). The procedure was identical to the study 3. Students received a link to the online questionnaire and access passwords to complete the questionnaire, after providing informed consent. At the beginning participants filled out the WEMWBS and next the Health Behaviours Inventory (HBI). The data was collected over a 1 month period, in June 2020.

#### Measures

##### The Warwick–Edinburgh Mental Wellbeing Scale

The Polish version of WEMWBS tested in the study 1 was used to assess mental wellbeing. The results of CFA confirmed that the one-factor solution was a very good fit to the data: (*χ2* (*77*) = 99.46; *p* = 0.043*; χ2/df* = 1.29; GFI = 0.98, CFI = 0.99, RMSEA = 0.066 [0.005 – 0.040]).

##### Pro-health behaviours

Pro-health behaviours were measured with the Health Behaviours Inventory (HBI) created by Juczyński (29). The HBI consists of 24 items describing different behaviours related to health (e.g. preventive behaviours, proper eating habits and health practices). The respondents estimated the frequency of particular health practices on the scale from 1 (almost never) to 5 (almost always). The scores range from 24 to 120. The results are converted into sten scores. Sten scores of 1–4 are regarded as low, 5–6 as average, and 7–10 as high. The internal consistency was good—Cronbach's alpha (0.85) [42].

#### Statistical analysis

First, we divided the sample based on sten scores into two groups: group with high level of pro-health behaviours (n = 57) and group with low level of pro-health behaviours (n = 215). Then we used Student’s *t-*test to examine the differences in wellbeing between groups. Effect sizes were evaluated with Cohen’s *d*.

### Results

We expected that group with high level of health-related behaviours would score higher on mental wellbeing than group with low level of health-related behaviour. The results of Student’s *t* test demonstrated significant differences in the level of mental wellbeing between groups (*t*(270) = − 7.44; *p* < 0.001; Cohen’s *d* = 1.17, a large effect). The participants with high scores on HBI showed a higher degree of mental wellbeing (*M* = 57.98, *SD* = 7.85) than participants with low scores on HBI (*M* = 47.72, *SD* = 9.57).

## Discussion

WEMWBS is a population measure of mental wellbeing, validated for use in adults aged 19 years and over in Poland. This study followed methods similar to those used for the original scale, to increase comparability. The Polish WEMWBS has been shown to be comprehensible and to provide reliable and consistent results. The initial goal of the study consisted of the empirical verification of the Polish adaptation of the WEMWBS questionnaire. The results presented confirm that the psychometric properties of the Polish adaptation of WEMWBS are very good. Using EFA and CFA it was shown that a one-factor solution is optimal. Support was found for the single factor hypothesis for scale. Furthermore, the high discriminatory power of the test items indicates high internal compliance. When compared to the validation of the original tool, the comparative analysis confirmed similar values for the model fit measures. Based on the results obtained, it has been shown that the adapted study obtained convergent factor validity like the original validation. Similar results, which confirmed a one-factor solution, have been indicated in other adaptive studies [10, 20, 21, 23]. Reliability, measured using the Cronbach’s alpha coefficient and McDonald's omega proved to be very high. The value obtained for the reliability coefficient confirms the possibility of using the WEMWBS questionnaire in both scientific research and mental well-being monitoring. Cronbach’s alpha and McDonald's omega were higher than the value obtained in the original validation. The average for the WEMWBS overall result was *M* = 49.87 (*SD* = 9.88). This result is lower than the average obtained in the original validation of the questionnaire, where the average was *M* = 50.7 (*SD* = 8.79).

The estimation of the absolute stability (test–retest) of the Polish version of the WEMWBS proved to be high. Similar reliability coefficients were obtained, among others, for the Chinese [23], Italian [21] and Spanish [25] adaptive tests as well as the original version [7, 18], The study confirmed also validity of the WEMWBS in Polish sample. The results for the WEMWBS were significantly related to life satisfaction and depression risk assessment. The relationship between mental wellbeing and life satisfaction was strong and positive, while between mental wellbeing and depression risk assessment strong and negative.

Our validation studies also provided data demonstrating sensitivity of the WEMWBS to detect a population with different levels of health-related behaviours, indicating that group with high level of pro-health behaviours achieved higher WEMWBS wellbeing results than group with low level of pro-health behaviours.

### Limitations

Presented Polish adaptation of the WEMWBS has several limitations. The further study with a more robust sample size is necessary to confirm the findings. The different clinical groups should be included to provide evidence for the broad application of WEMWBS. The high percentage of young participants is limitation of the external validity of our study and thus future research should be conducted with a general population sample. Furthermore, the research presented in this article is correlative in nature, and the collected data come only from self-descriptions, so caution is needed when interpreting the results. To avoid this limitation, experimental methods and data that are not self-descriptive should be used in future research. For the validation process this is not of major relevance, but for future studies a more balanced method is required.

## Conclusions

WEMWBS was confirmed as a short, reliable and valid measure with good psychometric properties and a correlation with life satisfaction and risk of depression. Therefore, WEMWBS has been proved to be an accurate and reliable tool for measuring the level of mental wellbeing. It seems that the scale can be useful for both individual research and intercultural and inter-group comparisons. Due to the high indicators for its psychometric properties, the scale may therefore prove to be a particularly useful tool not only in empirical research, but also in monitoring of mental wellbeing, and could serve as support in educational, preventive or social activities. According to data presented in this paper, which were derived from the process of adapting the scale in Poland, the psychometric parameters of the scale are good and similar to those obtained by the authors in earlier adaptation studies [21, 22, 44].

## Data Availability

The data that support the findings of this study are available from https://doi.org/10.3886/E120071V1.

## Abbreviations

M: Mean
SD: Standard deviation
EFA: Exploratory factor analysis
CFA: Confirmatory factor analysis
CFI: Comparative fit index
RMSEA: Root mean squared error of approximation
CFI: Comparative fit index
GFI: Goodness-of-fit index
ICC: Intra-class correlation coefficient
IDI: Item Difficulty Index
WEMWBS: Warwick–Edinburgh Mental Wellbeing Scale
WHO: World Health Organisation
HBI: Health Behaviours Inventory
KADS: Kutcher Adolescent Depression Scale
SWLS: Satisfaction with Life Scale
PANAS: Positive and Negative Affect Schedule
PWBS: Psychological Well-Being Scale
PA: Parallel analysis
DWLS: Diagonally weighted least squares
